# Prognostic Utility of Platelet-to-Albumin Ratio among Critically Ill Patients with Colorectal Cancer: A Propensity Score Matching Study

**DOI:** 10.1155/2022/6107997

**Published:** 2022-05-26

**Authors:** Anshu Li, Zhiyong Wang, Qing Lv, Yan Ling

**Affiliations:** ^1^Department of Gastrointestinal Surgery, Union Hospital, Tongji Medical College, Huazhong University of Science and Technology, 430022 Wuhan, China; ^2^Health Management Center, Union Hospital, Tongji Medical College, Huazhong University of Science and Technology, 430022 Wuhan, China

## Abstract

The platelet-to-albumin ratio (PAR) was developed to evaluate inflammatory and nutritional status among patients. The primary goal of the current study was to gain insight into the prognostic role of PAR in critically ill patients with colorectal cancer (CRC). The secondary aim was to develop and verify a clinical model including PAR for the prediction of 28-day mortality. This observational, multicenter study used data from the Medical Information Mart for Intensive Care (MIMIC) IV, e-ICU databases, and Union cohort. Data from 776 critically ill patients with CRC were from the e-ICU database, 219 from the MIMC-IV database, and 135 from the Wuhan Union Hospital. Propensity score matching (PSM) analysis, along with inverse probability treatment weighting, was used to control the influence of confounding factors. Support vector machine (SVM) and LASSO Cox models were then applied to identify significant metrics associated with 28-day mortality in the test cohort. Receiver operating curve (ROC) analysis, along with sensitivity and specificity, was measured to assess the predictive performances of PAR and the survival nomogram. The threshold value for PAR was 8.6, and patients with high PAR (≥8.6) experienced higher 28-day mortality compared to those with low PAR (<8.6). ROC curve analyses revealed that the discriminative ability of PAR was better than platelet count and albumin alone. LASSO Cox regression along with SVM identified six significant metrics associated with 28-day mortality in critically ill patients with CRC, including PAR. The C-index of the critically ill CRC nomogram was 0.802 (0.744–0.859) in the e-ICU training cohort, 0.839 (0.779–0.899) in the e-ICU validation cohort, 0.787 (0.695–0.879) in the MIMIC-IV cohort, and 0.767 (0.703–0.831) in the Union cohort. PAR is a simple score that combines inflammatory and nutritional status. PAR was a reliable index to predict short-term survival outcome of critically ill patients with CRC. Moreover, a clinical nomogram incorporating PAR exhibited satisfactory performance for predicting 28-day mortality of critically ill patients with CRC.

## 1. Introduction

Colorectal cancer (CRC) is reported to be the third most common cancer in terms of incidence and the second leading cause of cancer-related death globally [1]. Despite major advances in CRC healthcare, the worldwide incidence and mortality rates of CRC continue to increase, with >2.2 million newly diagnosed cases and 1.1 million deaths projected by 2030 [2]. Major progress has been achieved in the treatment of CRC in past decades, especially in the field of molecular targeted therapy and immunotherapy. Nevertheless, a vast number of CRC patients with advanced stage disease do not benefit from these therapies, and their long-term survival outcomes remain unsatisfactory [3].

Most recent research has focused on biomarkers for the early diagnosis of CRC and the assessment of long-term survival outcomes of individuals with CRC. However, few studies have specifically devoted attention to critically ill patients with CRC. Advances in anticancer treatment and survival evaluation are closely linked to the increased number of patients with CRC requiring intensive care [4]. CRC patients in advanced stages of disease are especially vulnerable to complications with severe infection, acute respiratory failure, cardiovascular events, and neurological disorders; thus these individuals commonly require intensive care [5]. Recently, mortality rates of CRC patients have decreased with the wide application of advanced organ support techniques. Because early organ support is related to improved survival for critically ill CRC patients, identification of novel biomarkers with adequate predictive accuracy is crucial for accurate risk stratification to avoid delayed organ support for individuals with CRC at high risk for death.

Inflammation and malnutrition are the important factors responsible for disease progression among patients with CRC [6]. The inflammatory response drives the progression of malnutrition, and continued malnutrition status may, in turn, induce severe and systemic inflammatory responses, which results in a vicious cycle [7]. In recent years, many oncologists have preferred to focus on clinical metrics combining malnutrition and inflammation. Sugimachi et al [8] investigated the significance of the immunonutritional index in evaluating the risk associated with the elderly patients undergoing pancreatectomy and found that immunonutritional status was remarkably impaired. Hayama et al [9] developed a nutrition inflammation status model based on cholesterol, serum albumin, neutrophil count, C-reactive protein (CRP), and platelet count for the prediction of overall survival among individuals with CRC. Liu et al. [10] also created a survival nomogram based on several immunonutritional indexes, and this immunonutritional model demonstrated good accuracy for the prediction of survival outcomes among CRC patients. Matsubara et al. [11] identified CRP-to-albumin ratio as the most significant prognostic biomarker among immunonutritional indexes among patients with non-small cell lung cancer. However, these studies were all based on well-established indexes, and no additional novel biomarkers were explored.

Platelet-to-albumin ratio (PAR), a combined indicator of nutritional and inflammatory status, has been indicated as a potent survival biomarker in peritoneal dialysis and various cancer. We hypothesized that PAR is correlated with the short-term mortality of critically ill patients with CRC. Therefore, we performed this clinical study to determine whether PAR could be a prognostic metric for critically ill patients with CRC in intensive care unit (ICU) through a propensity score matching (PSM) analysis. Then, we will design and validate a clinical model consisting of PAR for the prediction of short-term mortality of critically ill patients with CRC.

## 2. Materials and Methods

### 2.1. Study Design

This is a retrospective and observational study based on data from two large critical databases (e-ICU and MIMIC-IV). The two databases mainly contain participants who are critically ill, but also contain individuals with cancer. E-ICU database was mined to design and internally verify the prognostic significance of PAR and survival nomogram, and MIMIC-IV database was searched to externally validate the significance of PAR and survival nomogram. Finally, we also collected the clinical data of 135 CRC patients who were admitted to ICU from Wuhan Union Hospital. Our study design was strictly in line with the Declaration of Helsinki, and our research plan was approved by the clinical ethics committee of Wuhan Union Hospital.

### 2.2. Data Collection

Data from critically ill patients with CRC were collected from two critical care databases; however, potentially eligible participants were excluded for the following reasons: complicated with other malignant tumors; lost or absent critical clinical information, such as platelet count, albumin, or survival data; and age <18 years. Ultimately, data from 776 critically ill patients with CRC were collected from the e-ICU database and 219 from the Medical Information Mart for Intensive Care (MIMIC) IV database. The main outcome of this retrospective analysis was 28-day, all-cause mortality of critically ill patients with CRC. The optimal cut-off value for PAR was determined using X-tile version 3.6.1 based on 28-day mortality. X-tile is an easy-to-use tool for the selection of survival outcome-based cut-point [12]. The X-tile software for grouping uses each number between the range of the removed PAR counts as the cut-off value. Subsequently, the *X*^2^ score and *P* value are measured using the number as the cut-off value. The final number with the maximum *X*^2^ score and the minimum *P* value was identified as the optimal cut-off value [13].

### 2.3. Statistical Analysis

Propensity score matching (PSM) and propensity score-based inverse probability of treatment weighing (IPTW) were also used to adjust the covariates to ensure robustness of the results. One-to-one nearest neighbor matching, with a caliper width of 0.05, was applied in the current study. An IPTW model was constructed using the estimated propensity scores as weights. Standardized mean differences were calculated to evaluate the effectiveness of the PSM and IPTW models. Continuous metrics are summarized as mean and standard deviation, and categorical indexes are expressed as frequency with percentage. Differences in clinical features between the high and low PAR groups were detected using the chi-squared test or Student's *t*-test according to the data type. Because machine learning methods could help to handle nonlinear and high-order terms automatically and improve the predictive performance of clinical model [14, 15], the support vector machine algorithm, along with LASSO Cox regression, was then applied to identify significant metrics associated with 28-day mortality in the test cohort, and only informative metrics with *P* < 0.05 were finally included in the construction of the survival nomogram. Receiver operating curve (ROC) along with sensitivity and specificity was measured to assess the predictive performances of PAR and survival nomogram. The Kaplan-Meier curves along with log-rank test were utilized to estimate the prognostic role of PAR and survival nomogram in critically ill patients with CRC. Sensitive analyses were conducted to enhance the robustness of our conclusions. All the statistical works were finished via SPSS software (version 25.0) and R software (4.1.0). *P* value no more than 0.05 implicates statistical significance.

## 3. Results

### 3.1. Description of Baseline Features

Based on the inclusion criteria, data from 776 critically ill patients with CRC from e-ICU database, 219 from the MIMIC-IV database, and 135 from the Union cohort were included. For the purpose of model construction and verification, subjects were randomly divided into an e-ICU cohort, a training cohort (*N* = 547), and an internal validation cohort (*N* = 229). Both the MIMIC-IV cohort (*N* = 219) and Union cohort (*N* = 135) served as external validation cohorts. As listed in Table S1, the mean age of the critically ill patients with CRC was 69.2 years in the e-IUC training cohort, 69.9 years in the e-ICU validation cohort, 69.4 years in MIMIC-IV cohort, and 57.5 years in Union cohort, indicating that older CRC patients were more likely to progress to critically ill status, probably due to underlying diseases or advanced tumor stage. The proportion of males was 57.0%, 63.3%, 57.5%, and 48.9% in the e-ICU training cohort, e-ICU validation cohort, MIMIC-IV cohort, and Union cohort, respectively. Regarding 28-day in-hospital mortality, there were no statistical differences among the four cohorts (13.3% in the e-ICU training cohort, 14.8% in the e-ICU validation cohort, 17.4% in the MIMIC-IV cohort, and 14.8% in the Union cohort).

**Figure 1:**
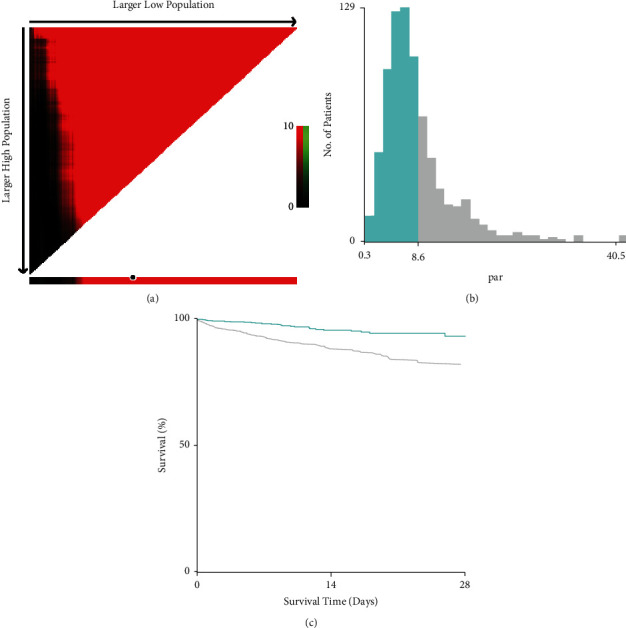
X-tile analyses of PAR values in e-ICU database. X-tile plots for critically ill patients with CRC (a). Black circles refers to the optimal threshold value of PAR (b). Kaplan-Meier curve of critically ill patients with CRC divided by PAR (c).

**Figure 2:**
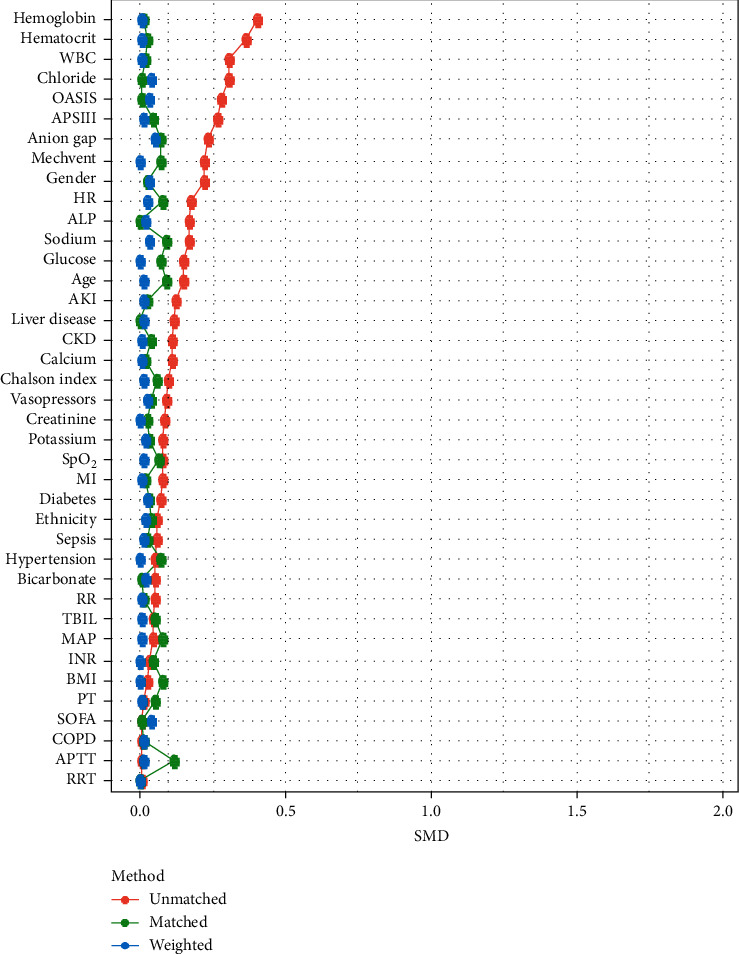
Standardized mean difference (SMD) of clinical metrics before and after propensity score matching and weighting in the e-ICU cohort.

Using 28-day in-hospital mortality as the final outcome, X-tile software was used to ascertain the threshold value of PAR. As shown in Figure 1, PAR demonstrated the most significant association with 28-day in-hospital mortality at a PAR value of 8.6. Subsequently, critically ill patients with CRC were divided into low and high PAR groups based on the PAR threshold value. In the e-ICU training cohort, there was a higher proportion of female CRC patients, use of mechanical ventilation, and higher Overall Anxiety Severity and Impairment Scale (OASIS) and Acute Physiology Score (APS) III scores in the high PAR group. Due to differences in several clinical metrics between the low and high PAR groups, PSM was applied to balance the distribution of common features. In total, 202 individuals with low PAR were matched with 202 exhibiting high PAR. To further reduce the imbalance between the low and high PAR groups, IPTW was also performed (Figure 2). As shown in Table 1, all clinical metrics were deemed to be well balanced in the weighted cohort. Receiver operating characteristic (ROC) curve analysis was used to compare the predictive performance of PAR, platelet count, and albumin level for 28-day mortality in critically ill patients with CRC. As shown in Table S2, PAR showed the highest predictive performance, not only in the e-ICU cohort (area under the ROC curve [AUC] 0.789), but also in the MIMIC-IV cohort (AUC 0.75).

### 3.2. Survival Analysis and Sensitivity Analysis of PAR

**Figure 3:**
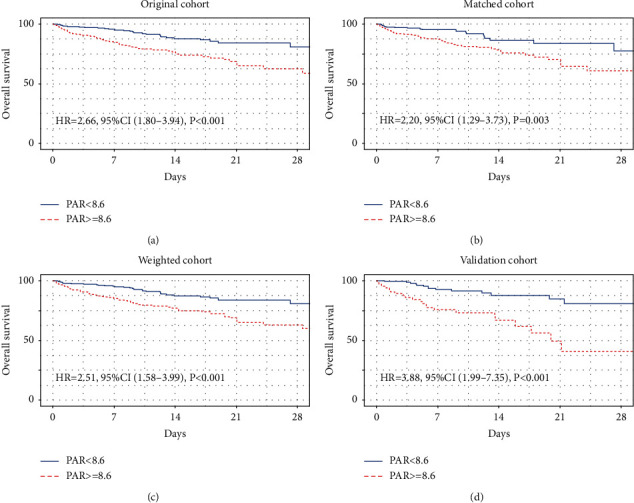
Kaplan-Meier curves for critically ill CRC patients stratified by PAR in the original cohort (a), in the matched cohort (b), in the weighted cohort (c), and in the validation cohort (d).

Kaplan-Meier curves comparing 28-day mortality of critically ill patients according to the cut-off for PAR are shown in Figure 3. CRC patients admitted to the ICU with high PAR demonstrated higher 28-day mortality compared to those with low PAR in the original cohort (hazard ratio [HR] 2.66 [95% confidence interval (CI) 1.80–3.94]; *P* < 0.0001) (Figure 3(a)). After balancing several confounding risk metrics, the strong correlation between 28-day mortality and high levels of PAR existed not only in the PSM cohort (HR 2.2 [95% CI 1.29–3.73]; *P*=0.003) (Figure 3(b)), but also in weighted cohort (HR 2.51 [95% CI 1.58–3.99]; *P* < 0.0001) (Figure 3(c)). Interestingly, when this survival correlation of PAR was validated using the same threshold value, CRC patients admitted to the ICU with high PAR experienced higher 28-day mortality compared to those with low PAR in the MIMIC-IV cohort (HR 3.88 [95% CI 1.99–7.35]; *P* < 0.0001) (Figure 3(d)). Regarding the sensitivity analysis, a univariate Cox model was constructed in the original, PSM, weighted, and validation cohorts to assess the predictive value of PAR for 28-day mortality. Some potential covariates were also adjusted in the three models, and the results demonstrated a similar tendency (*P* < 0.01) (Table 2).

### 3.3. Clinical Model for the Survival Prediction of Critically Ill Patients with CRC

**Figure 4:**
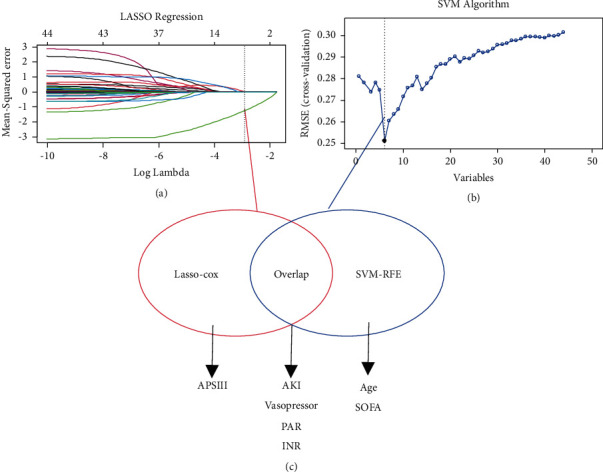
Selection of informative factors associated with 28-day mortality using the LASSO Cox regression model and SVM algorithm. (a) LASSO coefficient profiles of 44 clinical features. (b) Selection process of SVM algorithm. (c) Four significant indexes were selected by LASSO Cox regression model and SVM algorithm.

**Figure 5:**
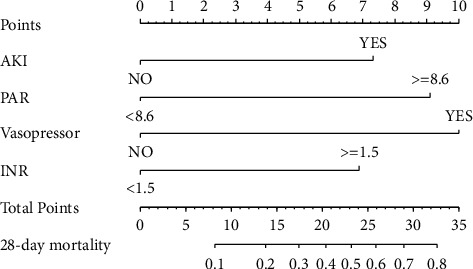
A clinical nomogram for the prediction of 28-day mortality among critically ill individuals with colorectal cancer.

The LASSO Cox model (Figure 4(a)), along with the SVM algorithm (Figure 4(a)), was used to identify clinical metrics closely associated with 28-day mortality of critically ill CRC individuals in the e-ICU training set. Final result (Figure 4(c)) revealed that the optimal survival model had four potential predictors (PAR, acute kidney injury, vasopressor, and international normalized ratio). Subsequently, the clinical nomogram was constructed for the prediction of 28-day mortality of critically ill patients with CRC based on the four potent risk factors (Figure 5). The four-factor clinical model was considered to be the critically ill CRC nomogram, and this clinical model achieved satisfactory predictive performance for the prediction of 28-day mortality in critically ill individuals with CRC. The C-index for the critically ill CRC nomogram was 0.873 (0.829–0.916) in the primary cohort, 0.896 (0.851–0.941) in the e-ICU validation cohort, 0.827 (0.743–0.912) in the MIMIC-IV cohort, and 0.767 (0.703–0.831) in the Union cohort.

### 3.4. Evaluation of the Clinical Model and Risk Stratification

**Figure 6:**
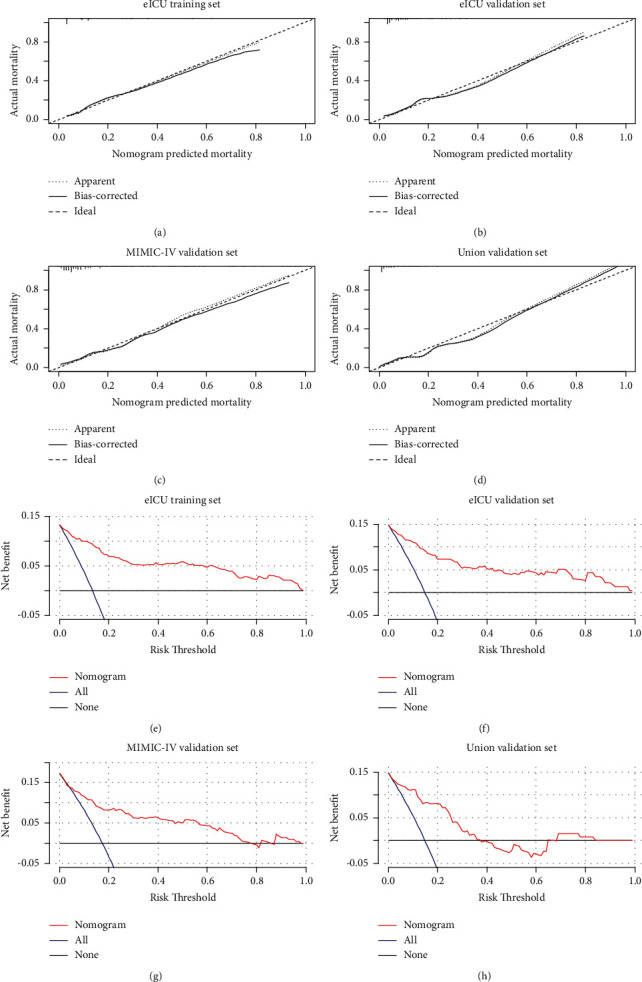
Calibration curves for in-hospital mortality in critically ill CRC patients in e-ICU training cohort (a), in e-ICU validation cohort (b), in MIMIC-IV cohort (c), and in the Union cohort (d). Decision curve analysis for 28-day mortality in CRC patients in ICU to detect its clinical usefulness in e-ICU training cohort (e), in e-ICU validation cohort (f), in MIMIC-IV cohort (g), and in the Union cohort (h).

Calibration curves were used to precisely measure the goodness of fit of the critically ill CRC nomogram. The critically ill CRC nomogram demonstrated an encouraging goodness of fit between the predicted and actual 28-day mortality among critically ill patients with CRC, not only in the e-ICU training (Figure 6(a)) and internal validation cohorts (Figure 6(b)), but also in the MIMIC-IV (Figure 6(c)) and Union (Figure 6(d)) cohorts. Decision curve analysis (DCA) was also implemented to assess the clinical utility of the critically ill CRC nomogram. As listed in Figures 6(e)–6(h), if the risk threshold of a critically ill CRC patient was 0.2, the critical ill CRC nomogram gained more clinical benefit than either treat-all regimen or treat-none scheme, suggesting that the survival nomogram was competent for the prediction of 28-day mortality among critically ill CRC patients in clinical practice.

**Figure 7:**
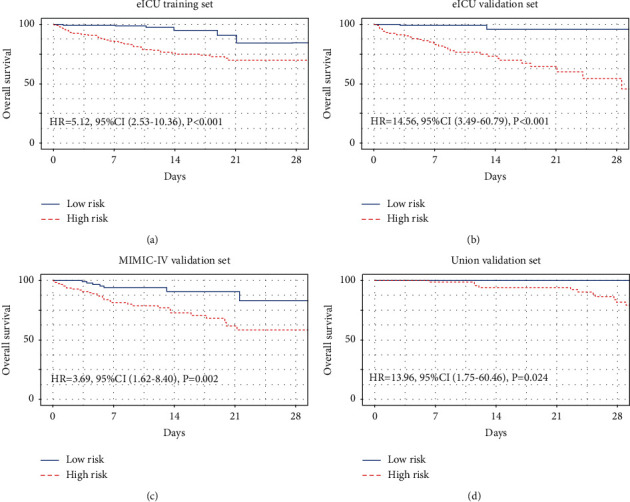
Kaplan-Meier curves of 28-day mortality for CRC patients stratified by the mean point predicted by the nomogram in e-ICU training cohort (a), in e-ICU validation cohort (b), in MIMIC-IV cohort (c), and in the Union cohort (d).

Finally, we divided critically ill CRC patients into the low risk and high risk subgroups based on the median value of the nomogram. A remarkably statistical difference in 28-day mortality between the low risk and high risk subgroups was revealed by Kaplan-Meier curve in the e-ICU training cohort (HR = 13.42, 95% CI: 4.88–36.91, *P* < 0.0001, Figure 7(a)), the e-ICU validation cohort (HR = 29.24, 95% CI: 3.7–59.24, *P*=0.004, Figure 7(b)), MIMIC-IV cohort (HR = 4.06, 95% CI: 1.68–9.77, *P*=0.002, Figure 7(c)), and Union cohort (HR = 13.96, 95% CI: 1.75–60.46, *P*=0.024, Figure 7(d)). Hence, the survival analyses revealed that the critically ill CRC nomogram could be used for risk stratification among critically ill CRC patients, and critically ill CRC patients with high risk might receive earlier and radical treatment.

## 4. Discussion

To our knowledge, the present investigation was the first clinical study based on two large cohorts to explore the association between PAR and survival outcome among critically ill patients with CRC and also confirmed the relationship with our own cohort. We found that higher PAR was correlated with an increased risk for 28-day mortality among critically ill patients with CRC, and PAR was a potent prognostic biomarker of short-term mortality after the adjustment for confounding variables. After PSM, we found the prognostic value of PAR for the prediction of 28-day mortality in critically ill patients with CRC was much higher compared to that before PSM. Moreover, we designed the first survival nomogram for the prediction of 28-day mortality in critically ill patients with CRC. This predictive model demonstrated good predictive performance not only in the e-ICU test and internal cohorts, but also in the MIMIC-IV and the Union cohorts, which are totally different from e-ICU database.

In past decades, platelet count was regarded to be a key factor in hemostasis and thrombosis in past decades. However, accumulating evidence has shown that platelets could contribute to tumorigenesis and invasion through complex crosstalk between platelets and tumor cells [16]. In the microenvironment of CRC, platelets can promote growth and metastasis of tumor cells via releasing transforming growth factor-beta and vascular epidermal growth factor. A recent study [17] reported that platelet count was positively correlated with serum levels of CRP and a variety of cytokines and highlighted the close correlation between platelets and inflammation status in CRC. Moreover, cytokines secreted by platelets can, in turn, promote cancer-associated inflammation [18].

On the other hand, albumin is an acute-phase protein used in clinical practice and decreases in response to inflammatory reactions and responses. Moreover, low levels of albumin generally signify malnutrition and can exert negative effects on survival outcomes among patients with CRC [19]. PAR, derived from platelet count and albumin levels, represents an entirely different index, combining both nutritional and inflammatory status. It is quite significant in simultaneously evaluating inflammatory and nutritional status in critically ill patients with CRC. High levels of PAR represent higher platelet counts with inflammatory response, and lower levels of albumin with malnutrition, eventually resulting in inferior short-term clinical outcomes of critically ill patients with CRC. ROC curve analysis revealed that PAR demonstrated better accuracy than platelet count or albumin in the prediction of 28-day mortality among critically ill patients with CRC. In addition, our analysis also demonstrated that ill patients with high PAR experienced higher 28-day mortality than those with low PAR.

Several clinical investigations have gained insight into the prognostic value of PAR. Yang et al. [20] performed a single-center study with 405 peritoneal dialysis patients and reported that PAR was a risk factor associated with mortality. Moreover, PAR not only contributed to critical illness but was also implicated in the risk assessment of a list of malignant tumors. Huang et al. [21] concluded that PAR was a potent risk factor for lymph node metastasis of gastric cancer and also constructed a clinical model including PAR for risk assessment of lymph node metastasis among individuals with gastric cancer. A recent study [22] involving 198 individuals with lung cancer revealed that high PAR was correlated with less unfavorable overall survival, while subsequent multivariate Cox analyses identified PAR as a potent risk indicator for worse overall survival. Saito et al. [23] assessed the prognostic role of PAR in cholangiocarcinoma and found that preoperative PAR was inversely associated with overall and disease-free survival in cholangiocarcinoma patients who underwent primary resection. Li et al. [24] reported that individuals with hepatocellular carcinoma with high preoperative PAR exhibited a lower long-term survival rate and higher recurrent risk than hepatocellular carcinoma patients with low PAR. However, no study has addressed the clinical association between PAR and mortality among critically ill patients with CRC. Consistent with the results from other cancers, survival analysis also demonstrated that critically ill CRC patients with high PAR exhibited a risk ratio of 2.66 for mortality compared with the low PAR group in the primary cohort; this ratio was increased to 3.88 in the validation cohort.

Our analysis had three primary limitations. First, we could not assess the relationship between PAR and long-term survival outcomes of critically ill individuals with CRC, such as six-month and one-year mortality due to technical reasons. Subsequently, we only abstracted baseline PAR on admission and did not have information about time variations in PAR. Finally, because the two databases only included data from critically ill patients, some important inflammatory and nutritional indexes were missing, such as prognostic nutritional index, CRP, and lactate dehydrogenase; as such, we could not appraise the relationship between PAR and these well-established inflammatory and nutritional indexes. Hence, multicenter trials including more established indexes and longer follow-up are needed to further validate the prognostic role of PAR in critically ill patients with CRC.

## 5. Conclusion

This was the first clinical analysis to investigate the prognostic role of PAR among critically ill patients with CRC. PAR is a simple score that combines inflammatory and nutritional status. PAR can be applied to predict short-term survival outcome of critically ill patients with CRC. Moreover, a survival nomogram incorporating PAR demonstrated satisfactory performance for predicting 28-day mortality in critically ill patients with CRC.

## Figures and Tables

**Table 1:** Comparison of baseline metrics between the low and high PAR groups.

Baseline metrics	Original cohort			Matched cohort			Validation cohort		
	Low PAR	High PAR	*P*	Low PAR	High PAR	*P*	Low PAR	High PAR	*p*
Number	511	265	—	202	202	—	154	65	—
Age, years	70.1 (13.4)	68.1(13.6)	0.051	68.6 (14.1)	69.5 (12.8)	0.472	69.7 (12.0)	68.7 (15.2)	0.620
Gender, male, *n* (%)	320 (62.6)	137 (51.7)	0.004	113 (55.9)	107 (53.0)	0.617	98 (63.6)	28 (43.1)	0.008
BMI, kg/m^2^	27.7 (7.9)	28.0 (8.3)	0.590	28.2 (8.4)	28.1 (8.3)	0.885	29.2 (8.4)	27.5 (8.3)	0.186

*Ethnicity, n (%)*			0.568			0.479			0.087
White	400 (78.3)	199 (75.1)		155 (76.7)	154 (76.2)		110 (71.4)	40 (61.5)	
Black	66 (12.9)	41 (15.5)		33 (16.3)	28 (13.9)		19 (12.3)	6 (9.2)	
Other	45 (8.8)	25 (9.4)		14 (6.9)	20 (9.9)		25 (16.2)	19 (29.2)	

*Interventions, n (%)*									
MV use	136 (26.6)	99 (37.4)	0.003	77 (38.1)	66 (32.7)	0.298	49 (31.8)	29 (44.6)	0.098
RRT use	6 (1.2)	3 (1.1)	1.000	1 (0.5)	1 (0.5)	1.000	7 (4.5)	0 (0.0)	0.185
Vasopressor use	75 (14.7)	48 (18.1)	0.093	33 (16.3)	32 (15.8)	1.000	57 (37.0)	23 (35.4)	0.940

*Score system, points*									
SOFA	3.5 (1.0)	3.4 (1.1)	0.909	3.6 (1.2)	3.4 (1.2)	0.592	4.8 (1.5)	5.4 (1.4)	0.282
OASIS	23.6 (10.2)	26.7 (10.4)	0.001	26.1 (10.9)	26.7 (10.1)	0.716	31.9 (8.7)	35.2 (9.8)	0.013
APSIII	43.5 (12.5)	50.0 (24.1)	0.001	48.6 (14.6)	47.7 (14.8)	0.725	47.8 (21.1)	56.1 (25.0)	0.021

*Comorbidities, n (%)*									
Hypertension	75 (14.7)	34 (12.8)	0.553	30 (14.9)	26 (12.9)	0.666	64 (41.6)	19 (29.2)	0.117
Diabetes	131 (25.6)	60 (22.6)	0.406	47 (23.3)	50 (24.8)	0.816	46 (29.9)	9 (13.8)	0.020
CKD	57 (11.2)	21 (7.9)	0.196	16 (7.9)	15 (7.4)	1.000	21 (13.6)	7 (10.8)	0.720
Myocardial infarct	41 (8.0)	16 (6.0)	0.390	16 (7.9)	16 (7.9)	1.000	25 (16.2)	6 (9.2)	0.252
CHF	64 (12.5)	20 (7.5)	0.046	24 (11.9)	15 (7.4)	0.178	35 (22.7)	11 (16.9)	0.434
COPD	63 (12.3)	32 (12.1)	1.000	22 (10.9)	23 (11.4)	1.000	30 (19.5)	18 (27.7)	0.245
Liver disease	11 (2.2)	2 (0.8)	0.253	1 (0.5)	1 (0.5)	1.000	16 (10.4)	4 (6.2)	0.461
CCI, points	5.7 (2.3)	5.4 (1.5)	0.191	5.5 (2.3)	5.5 (2.5)	0.965	8.9 (2.6)	9.0 (2.9)	0.670

*Complications, n (%)*									
AKI	119 (23.3)	81 (30.6)	0.168	54 (26.7)	49 (24.3)	0.347	43 (27.9)	20 (30.8)	0.793
Sepsis	76 (14.9)	45 (17.0)	0.507	29 (14.4)	28 (13.9)	1.000	73 (47.4)	29 (44.6)	0.818

*Vital signs*									
MAP, mmHg	87.1 (16.1)	87.9 (17.4)	0.548	88.9 (16.0)	87.7 (17.0)	0.464	83.0 (19.6)	81.6 (16.7)	0.623
Heart rate, bpm	89.2 (21.0)	93.9 (21.7)	0.004	92.4 (22.1)	92.2 (22.4)	0.934	96.5 (24.3)	100.6 (23.2)	0.248
RR, bpm	19.1 (5.0)	19.3 (5.4)	0.504	19.6 (5.7)	19.6 (5.4)	0.943	19.9 (5.4)	20.8 (6.3)	0.252
SpO2, %	96.9 (3.2)	96.6 (4.7)	0.080	96.6 (4.0)	96.7 (3.4)	0.687	95.8 (5.7)	97.0 (4.7)	0.137

*Laboratory results*									
WBC, ×10^9^/L	10.5 (4.0)	13.5 (5.8)	0.001	12.3 (4.8)	12.1 (4.7)	0.817	11.2 (4.3)	13.4 (4.8)	0.123
HGB, g/dL	11.2 (2.3)	10.1 (2.3)	0.001	10.4 (2.3)	10.4 (2.2)	0.898	10.4 (1.9)	9.8 (2.2)	0.033
PLT, ×10^9^/L	186.7 (70.9)	388.2 (95.7)	0.001	195.1 (77.8)	370.9 (97.6)	<0.001	189.0 (64.3)	346.9 (94.9)	<0.001
HCT, %	34.1 (6.7)	31.5 (6.6)	0.001	32.3 (6.9)	32.3 (6.2)	0.973	32.5 (5.6)	31.0 (5.9)	0.070
Albumin, g/dL	3.5 (0.7)	2.9 (0.6)	0.001	3.5 (0.7)	2.9 (0.7)	<0.001	3.5 (0.6)	2.7 (0.7)	<0.001
PAR	5.4 (1.9)	14.0 (5.0)	0.001	5.6 (1.9)	13.4 (5.5)	<0.001	5.5 (1.8)	13.0 (4.0)	<0.001
ALP, U/L	138.5(64.9)	174.3 (72.7)	0.018	169.5 (65.4)	154.8(61.5)	0.501	132.7(68.90	132.1(67.2)	0.965
Bilirubin, mmol/L	1.2 (0.5)	1.3 (0.6)	0.504	1.3 (0.5)	1.2 (0.5)	0.612	1.6 (0.6)	1.1 (0.5)	0.217
Anion gap, mEq/L	10.8 (4.0)	12.0 (4.9)	0.001	11.5 (4.6)	11.2 (4.3)	0.445	14.7 (4.2)	15.5 (4.7)	0.221
Bicarbonate, mEq/L	24.0 (4.5)	24.2 (4.6)	0.504	24.2 (4.6)	24.4 (4.2)	0.569	22.8 (4.6)	22.6 (5.4)	0.783
BUN, mg/dL	22.9 (8.2)	24.9 (9.9)	0.166	24.1 (9.4)	23.1 (8.4)	0.593	24.2 (10.8)	25.6 (12.6)	0.662
Creatinine, mg/dL	1.3 (0.5)	1.4 (0.6)	0.254	1.4 (0.5)	1.4 (0.5)	0.628	1.3 (0.6)	1.2 (0.5)	0.572
Glucose, mg/dL	141.0(63.2)	131.4(50.2)	0.032	140.0(67.3)	133.1 (52.2)	0.249	144.9 (59.3)	133.5 (56.0)	0.189
Potassium, mmol/L	4.1 (0.7)	4.2 (0.9)	0.269	4.1 (0.9)	4.2 (0.8)	0.521	4.2 (0.9)	4.2 (0.7)	0.796
Sodium, mmol/L	137.2 (5.3)	136.2 (5.9)	0.022	136.2(6.2)	136.4(5.6)	0.783	137.4 (5.2)	137.8 (4.1)	0.579
Calcium, mg/dL	8.5 (1.1)	8.6 (0.9)	0.160	8.6 (1.0)	8.6 (0.9)	0.812	8.4 (0.7)	8.2 (0.6)	0.216
Chloride, mmol/L	103.3 (6.3)	101.4 (6.8)	0.001	101.8 (7.0)	102.1 (6.3)	0.725	102.7 (7.1)	102.3 (5.4)	0.704
PT, s	16.6 (7.8)	16.8 (7.3)	0.851	17.2 (7.3)	16.5 (7.4)	0.480	15.7 (5.3)	16.2 (5.7)	0.484
APTT, s	34.9 (10.7)	35.0 (8.0)	0.933	35.7 (11.6)	34.8 (7.8)	0.480	36.4 (10.8)	36.7 (11.7)	0.903
INR	1.5 (0.5)	1.5 (0.5)	0.686	1.5 (0.6)	1.5 (0.5)	0.577	1.4 (0.5)	1.5 (0.5)	0.526

*Clinical outcome*									
LOS, days	10.9 (4.6)	12.2 (5.2)	0.109	12.2 (5.4)	12.0 (5.4)	0.838	11.7 (5.6)	10.9 (5.3)	0.606
Death, *n* (%)	42 (8.2)	65 (24.5)	0.001	20 (9.9)	55 (22.3)	0.001	15 (9.7)	23 (35.4)	<0.001

**Table 2:** Summary of results of 28-day mortality and sensitivity analysis.

	Original cohort		Matched cohort		Weighted cohort		Validation cohort	
	HR (95% CI)	*P* value	HR (95% CI)	*P* value	HR (95% CI)	*P* value	HR (95% CI)	*P* value
Unadjusted	2.66 (1.80–3.94)	<0.001	2.20 (1.29–3.73)	0.003	2.51 (1.58–3.99)	<0.001	3.82 (1.99–7.35)	<0.001
Model 1	2.74 (1.85–4.08)	<0.001	2.24 (1.31–3.84)	0.007	2.54 (1.57–4.11)	<0.001	3.77 (1.86–7.64)	<0.001
Model 2	3.07 (2.03–4.64)	<0.001	2.81 (1.59–4.97)	<0.001	2.79 (1.71–4.55)	<0.001	4.59 (2.04–10.34)	<0.001
Model 3	3.44 (2.15–5.49)	<0.001	3.81 (1.98–7.35)	<0.001	3.73 (2.02–6.90)	<0.001	6.79 (2.58–17.87)	<0.001

Model 1 adjusted for age, gender, BMI, ethnicity. Model 2 adjusted for model 1 plus comorbidities and Charlson comorbidity index. Model 3 adjusted for model 2 plus score system, interventions, complications.

## Data Availability

The datasets used during the current study are available from the corresponding author on reasonable request.

## Abbreviation

CRC: Colorectal cancer
PAR: Platelet-to-albumin ratio
BMI: Body mass index
MV: Mechanical ventilation
RRT: Renal replacement therapy
SOFA: Sequential organ failure assessment
OASIS: Oxford acute severity of illness score
APSII: Acute physiology score III
CKD: Chronic kidney disease
CHF: Congestive heart failure
COPD: Chronic obstructive pulmonary disease
CCI: Charlson comorbidity index
AKI: Acute kidney injury
MAP: Mean arterial pressure
RR: Respiratory rate
WBC: White blood cell
HGB: Hemoglobin
PLT: Platelet
HCT: Hematocrit
ALP: Alkaline phosphatase
BUN: Blood urea nitrogen
PT: Prothrombin time
APTT: Activated partial thromboplastin time
INR: International normalized ratio
LOS: Length of hospital
SVM: Support vector machine
PTW: Probability of treatment weighing
PSM: Propensity score matching.

## Supplementary Materials

Supplementary Materials Table S1. Comparisons of baseline characteristics among three cohorts. Table S2. ROC analysis of PAR, platelet count, and albumin.
