# Supplementary material for: Prognostic Utility of Platelet-to-Albumin Ratio among Critically Ill Patients with Colorectal Cancer: A Propensity Score Matching Study
Source: J Oncol. 2022 May 26;2022:6107997. doi: 10.1155/2022/6107997 (PMC9162859; doi:10.1155/2022/6107997)
Supplement: Supplementary Materials — Table S1. Comparisons of baseline characteristics among three cohorts. Table S2. ROC analysis of PAR, platelet count, and albumin. [file 6107997.f1.doc]

Table S1. Comparisons of baseline characteristics among three cohorts

| Clinical metrics | E-ICU training set | E-ICU validation set | MIMIC-IV set | Union set | P value |
| --- | --- | --- | --- | --- | --- |
| Number | 547 | 229 | 219 | 135 | - |
| Age, years | 69.2 (13.4) | 69.9 (13.8) | 69.4 (13.0) | 57.5 (11.8) | <0.001 |
| Gender, male, n (%) | 312 (57.0) | 145 (63.3) | 126 (57.5) | 66 (48.9) | 0.078 |
| BMI, kg/m^2^ | 27.3 (7.9) | 27.0 (7.4) | 28.7 (8.4) | 22.9 (2.8) | 0.549 |
| Ethnicity, n (%) |  |  |  |  | <0.001 |
| White | 417 (76.2) | 182 (79.5) | 150 (68.5) | 0 (0.0) |  |
| Black | 75 (13.7) | 32 (14.0) | 25 (11.4) | 0 (0.0) |  |
| Other | 55 (10.1) | 15 (6.5) | 44 (20.1) | 135 (100.0) |  |
| Interventions, n (%) |  |  |  |  |  |
| MV use | 165 (30.2) | 70 (30.6) | 78 (35.6) | 87 (64.4) | <0.001 |
| RRT use | 7 (1.3) | 2 (0.9) | 7 (3.2) | 1 (0.1) | 0.058 |
| Vasopressor use | 89 (16.3) | 34 (14.8) | 80 (36.5) | 25 (18.5) | <0.001 |
| Score system, points |  |  |  |  |  |
| SOFA | 3.4 (1.7) | 3.6 (1.2) | 4.9 (2.1) | 4.3 (1.3) | <0.001 |
| OASIS | 24.7 (10.4) | 24.6 (10.3) | 32.9 (9.2) | 27.9 (9.6) | <0.001 |
| APSIII | 45.5 (22.9) | 46.2 (12.0) | 50.3 (24.3) | 51.4 (23.6) | 0.011 |
| Comorbidities, n (%) |  |  |  |  |  |
| Hypertension | 85 (15.5) | 24 (10.5) | 83 (37.9) | 46 (34.1) | <0.001 |
| Diabetes | 137 (25.0) | 54 (23.6) | 55 (25.1) | 32 (23.7) | 0.965 |
| CKD | 52 (9.5) | 26 (11.4) | 28 (12.8) | 15 (11.1) | 0.586 |
| Myocardial infarct | 45 (8.2) | 12 (23.6) | 31 (14.2) | 15 (11.1) | 0.007 |
| CHF | 65 (11.9) | 19 (8.3) | 46 (21.0) | 12 (8.9) | <0.001 |
| COPD | 66 (12.1) | 29 (12.7) | 48 (21.9) | 27 (20.0) | 0.001 |
| Liver disease | 11 (2.0) | 2 (0.9) | 20 (9.1) | 9 (6.7) | <0.001 |
| CCI, points | 5.5 (2.3) | 5.9 (2.5) | 8.9 (2.6) | 7.6 (2.6) | <0.001 |
| Complications, n (%) |  |  |  |  |  |
| AKI | 137 (25.0) | 63 (37.5) | 63 (28.8) | 29 (21.5) | 0.027 |
| Sepsis | 86 (15.7) | 35 (15.3) | 102 (46.6) | 38 (28.1) | <0.001 |
| Vital signs |  |  |  |  |  |
| MAP, mmHg | 87.7 (17.1) | 86.3 (16.3) | 82.6 (18.8) | 85.5 (21.2) | 0.004 |
| Heart rate, bpm | 91.0 (21.6) | 90.5 (20.8) | 97.7 (24.0) | 103.2 (24.3) | <0.001 |
| RR, bpm | 19.2 (5.3) | 19.0 (4.7) | 20.2 (5.7) | 21.7 (6.0) | <0.001 |
| SpO2, % | 96.8 (4.1) | 96.7 (3.0) | 96.2 (5.4) | 96.7 (5.1) | 0.786 |
| Laboratory results |  |  |  |  |  |
| WBC, × 10^9^/L | 11.6 (4.5) | 11.1 (4.0) | 11.9 (4.9) | 5.8 (1.7) | <0.001 |
| HGB, g/dL | 10.8 (2.4) | 10.8 (2.3) | 10.2 (2.0) | 36.2 (6.5) | <0.001 |
| PLT, × 10^9^/L | 255.1 (67.3) | 257.0 (66.0) | 236.2 (70.4) | 229.1 (70.8) | 0.049 |
| HCT, % | 33.2 (6.9) | 33.2 (6.5) | 32.0 (5.7) | 36.2 (6.5) | <0.001 |
| Albumin, g/dL | 3.3 (0.8) | 3.2 (0.7) | 3.3 (0.7) | 4.0 (0.5) | <0.001 |
| PAR | 8.3 (2.5) | 8.5 (2.7) | 7.7 (2.4) | 7.2 (2.5) | 0.057 |
| ALP, U/L | 149.2 (63.3) | 155.0 (64.2) | 133.4 (51.2) | 73.4 (21.1) | <0.001 |
| Bilirubin, mmol/L | 1.2 (0.4) | 1.4 (0.5) | 1.4 (0.6) | 0.7 (0.2) | 0.013 |
| Anion gap, mEq/L | 11.2 (4.3) | 11.1 (4.7) | 14.9 (4.3) | - | <0.001 |
| Bicarbonate, mEq/L | 24.0 (4.4) | 24.4 (4.8) | 22.7 (4.8) | 24.7 (6.3) | <0.001 |
| BUN, mg/dL | 23.8 (9.4) | 23.1 (7.3) | 24.6 (7.3) | 24.9 (7.1) | 0.532 |
| Creatinine, mg/dL | 1.3 (0.6) | 1.3 (0.4) | 1.3 (0.4) | 0.8 (0.2) | <0.001 |
| Glucose, mg/dL | 139.4 (61.3) | 135.2 (54.0) | 141.4 (58.4) | 132.6 (56.8) | 0.463 |
| Potassium, mmol/L | 4.1 (0.8) | 4.1 (0.7) | 4.2 (0.8) | 4.1 (0.8) | 0.656 |
| Sodium, mmol/L | 137.6 (5.5) | 137.1 (5.5) | 137.5 (4.9) | 137.6 (4.7) | 0.142 |
| Calcium, mg/dL | 8.5 (0.9) | 8.5 (0.9) | 8.3 (0.7) | 8.3 (0.7) | <0.001 |
| Chloride, mmol/L | 103.7 (6.6) | 103.4 (6.3) | 103.6 (6.6) | 102.9 (6.6) | 0.781 |
| PT, s | 16.4 (6.7) | 17.3 (7.3) | 15.8 (5.4) | 14.4 (0.8) | 0.002 |
| APTT, s | 35.1 (10.6) | 34.6 (7.8) | 36.5 (10.1) | 36.2 (3.8) | 0.314 |
| INR | 1.5 (0.7) | 1.5 (0.5) | 1.4 (0.5) | 1.0 (0.2) | <0.001 |
| Clinical outcome |  |  |  |  |  |
| LOS, days | 11.3 (4.9) | 11.3 (5.0) | 11.4 (5.8) | 27.9 (9.1) | <0.001 |
| Death, n (%) | 73 (13.3) | 34 (14.8) | 38 (17.4) | 20 (14.8) | 0.566 |

Table S2. ROC analysis of PAR, platelet count and albumin

| Variable | Sensitivity | Specificity | AUC (95%CI) | P value |
| --- | --- | --- | --- | --- |
| Original cohort |  |  |  |  |
| PAR | 75.6 | 71.1 | 0.789 (0.763-0.813) |  |
| PLT | 24.8 | 82.8 | 0.523 (0.491-0.554) | <0.0001 |
| Albumin | 75.9 | 55.7 | 0.700 (0.670-0.728) | 0.0006 |
| Matched cohort |  |  |  |  |
| PAR | 81.5 | 70.8 | 0.803 (0.761-0.841) |  |
| PLT | 43.1 | 71.7 | 0.544 (0.494-0.593) | <0.0001 |
| Albumin | 77.6 | 62.9 | 0.717 (0.670-0.760) | 0.0020 |
| MIMIC-IV cohort |  |  |  |  |
| PAR | 74.7 | 70.7 | 0.750 (0.687-0.806) |  |
| PLT | 55.3 | 58.0 | 0.522 (0.454-0.590) | <0.001 |
| Albumin | 71.2 | 55.3 | 0.638 (0.571-0.703) | 0.046 |
| Union cohort |  |  |  |  |
| PAR | 70.0 | 71.7 | 0.736 (0.667-0.788) |  |
| PLT | 55.0 | 60.9 | 0.589 (0.504-0.656) | 0.037 |
| Albumin | 73.9 | 55.0 | 0.647 (0.560-0.727) | NS |

PAR:platelet to serum albumin ratio, PLT:platelet, AUC:area under the receiver operating curve.
